# Interactions of pixantrone with apurinic/apyrimidinic sites in DNA

**DOI:** 10.17912/micropub.biology.001207

**Published:** 2024-06-08

**Authors:** Irina G. Minko, Michael M. Luzadder, Amanda K. McCullough, R. Stephen Lloyd

**Affiliations:** 1 Oregon Institute of Occupational Health Sciences, Oregon Health & Science University, Portland, Oregon, United States; 2 Department of Molecular and Medical Genetics, Oregon Health & Science University, Portland, Oregon, United States

## Abstract

Pixantrone and mitoxantrone are structurally related anticancer drugs which have been shown to generate covalent conjugates at apurinic/apyrimidinic (AP) sites in DNA. Mitoxantrone binding to AP sites induces DNA strand cleavage and inhibits the endonuclease activity of human AP endonuclease 1 (APE1). Here, pixantrone was demonstrated to have similar properties, but relative to mitoxantrone, it was significantly less potent in both DNA incision and APE1 inhibition. Consistent with these observations, pixantrone had ~ 15-fold lower affinity for DNA containing an AP site analogue, tetrahydrofuran, as measured by a Thiazole Orange (ThO) displacement assay.

**Figure 1. Interactions of pixantrone with AP site-containing DNA:**
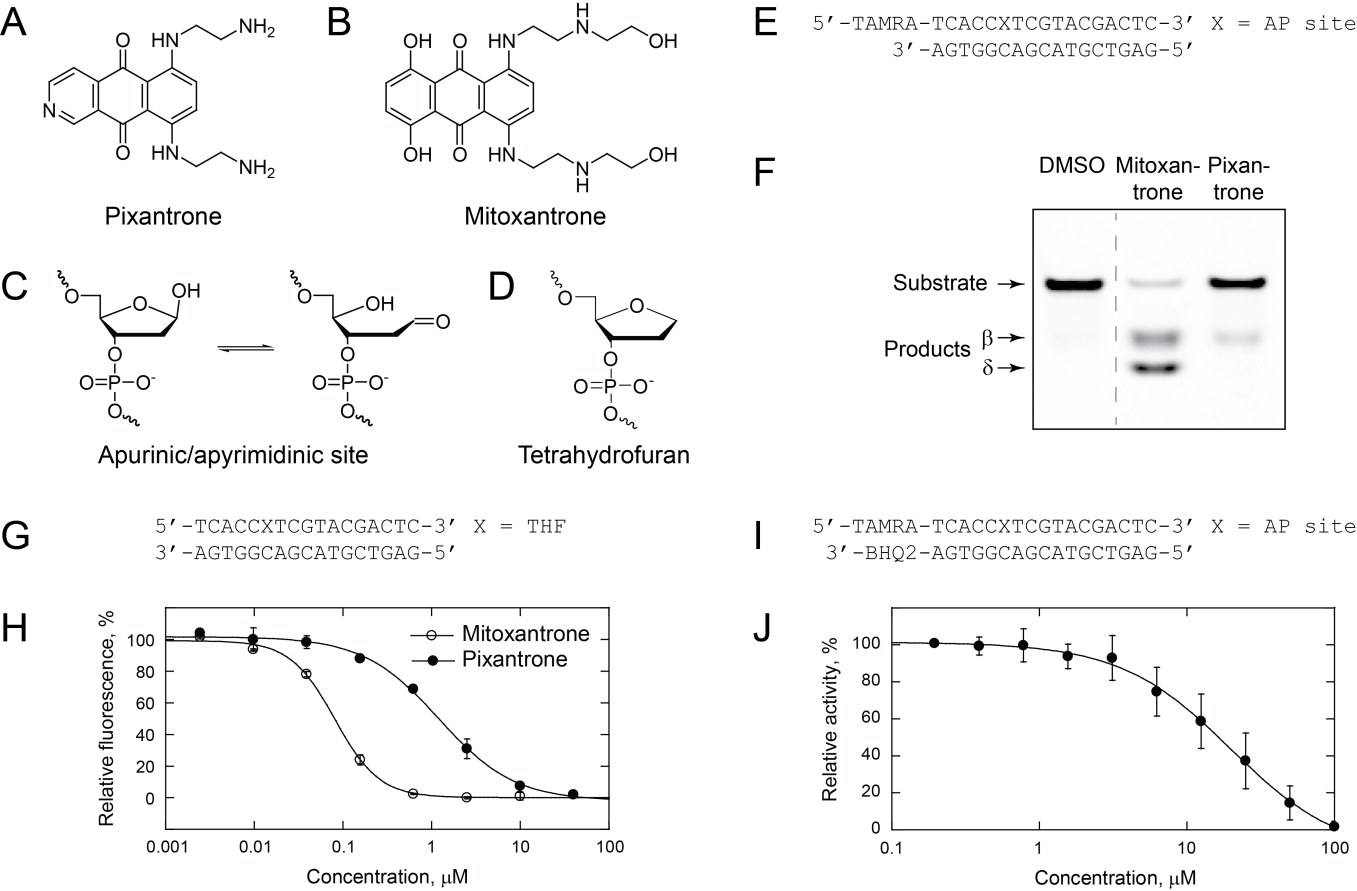
**A** . Pixantrone structure. **B** . Mitoxantrone structure. **C** . AP site structure shown as equilibrium between the ring-closed and ring-opened aldehyde form. **D** . Tetrahydrofuran structure. **E** . Oligodeoxynucleotides used in DNA incision assays. **F** . Incision of AP site-containing DNA. DNA (100 nM) was incubated with 10 μM mitoxantrone or pixantrone at 37 °C for 30 min. A representative gel is shown. **G** . Oligodeoxynucleotides used in the ThO dye displacement assay. **H** . ThO displacement by anthracenedione compounds: concentration-dependent decrease in ThO fluorescence. Mitoxantrone and pixantrone were incubated with 50 nM DNA and 250 nM ThO for 10 min at room temperature, and fluorescence recorded using a plate reader. The data were fit to a sigmoidal equation (correlation coefficient R > 0.99). **I** . Oligodeoxynucleotides used for IC _50_ measurement. **J** . Inhibition of APE1 activity on an AP site. AP site-containing DNA was preincubated with pixantrone at room temperature for 10 min and following addition of APE1, product formation was monitored at 37 °C in a plate reader. Reactions contained 50 nM DNA, 0.195 μM to 100 μM pixantrone, 0.00125 units/μl APE1, and 10 % (v/v) DMSO. To account for pixantrone-catalyzed cleavage of DNA substrate, reactions were also conducted using the same pixantrone concentrations, but in the absence of APE1. The control reference reactions contained no pixantrone. The initial rates of APE1-catalyzed DNA incision were calculated as described in Methods and plotted as a function of pixantrone concentrations. To obtain the IC _50_ value, the data were fitted to a sigmoidal equation (correlation coefficient R > 0.99).

## Description

Pixantrone (6,9-bis[(2-aminoethyl)amino] benzo[g]isoquinoline-5,10-dione dimaleate; Fig. 1A ) is an anthracenedione compound that has anticancer activity. Pixantrone was identified in a search for anthracenediones that lack the characteristic cardiotoxicities that are associated with this class of drug molecules, while retaining anticancer efficacy (Beggiolin et al., 2001; Cavalletti et al., 2007; Hasinoff et al., 2016) . Pixantrone induces DNA strand breaks through the poisoning of topoisomerase II reactions (Hasinoff et al., 2016; Mansour et al., 2022) . It also interacts with DNA via multiple binding modes, including intercalation into the double helix (De Isabella et al., 1995; Lima et al., 2019) . The same mechanisms of cytotoxicity have been established for mitoxantrone ( Fig. 1B ), a structurally related compound that is used in the treatment of certain types of solid tumors and hematologic malignancies (Boland et al., 2000; De Isabella et al., 1995; Evison et al., 2016; Lima et al., 2019) . In addition, DNA alkylation has been proposed as a mechanism for cell killing by these compounds, with adducts being formed at the exocyclic nitrogen atoms of guanines via formaldehyde-mediated aminal linkage (Evison et al., 2007; Evison et al., 2016; Mansour et al., 2022; Menna et al., 2016) .

Recently, it has been demonstrated that in addition to non-covalent interactions with DNA molecules and formation of formaldehyde-mediated DNA adducts, these anthracenediones can conjugate to apurinic/apyrimidinic (AP) sites in DNA (Bellamri et al., 2023) . The covalent linkage occurs between the ring-opened, aldehyde form of AP sites ( Fig. 1C ) and the primary or secondary amines in the aliphatic chains of pixantrone or mitoxantrone, respectively. Consistent with Schiff base chemistry, this linkage can be stabilized by reduction with sodium borohydride compounds (Bellamri et al., 2023; Dodson et al., 1993; Schrock and Lloyd, 1991) . Subsequent characterization of mitoxantrone-mediated conjugates by our group revealed that following formation of these complexes, β- and β,δ-elimination reactions can occur, creating a nick in DNA at an AP site (Minko et al., 2024) . Thus, mitoxantrone has an AP lyase activity. Additionally, it has been demonstrated that specific binding of mitoxantrone at AP sites and β-elimination products interferes with DNA repair functions of human AP endonuclease 1 (APE1) (Minko et al., 2024) . The mechanism of inhibition appeared to be independent of formation of the covalent linkage, since reduced endonuclease activity of APE1 in the presence of mitoxantrone was also observed on DNA containing tetrahydrofuran (THF) (Minko et al., 2024; Simeonov et al., 2009) , an AP site analogue that cannot undergo ring-opening and is not susceptible to Schiff base chemistry ( Fig. 1D ). Considering the structural similarity of mitoxantrone and pixantrone, interactions of pixantrone with AP site-containing DNA have been investigated in the present study.

To test for the ability of pixantrone to incise DNA at AP sites, TAMRA-labeled double-stranded oligodeoxynucleotides containing an AP site ( Fig. 1E ) were incubated with 10 μM pixantrone at 37 °C for 30 min and the products analyzed following separation by gel electrophoreses. These data demonstrated that pixantrone could catalyze a β-elimination reaction, with no evidence for the formation of the δ product ( Fig. 1F ). This was in contrast to mitoxantrone, which generated both the β and δ products. The quantity of products formed in the presence of 10 μM pixantrone was low, 12 ± 3 % versus 82 ± 3 % produced by 10 μM mitoxantrone. Prior analyses demonstrated that 1 μM mitoxantrone generated ~ 20 % of products under identical conditions (Minko et al., 2024) . Thus, pixantrone is approximately one order of magnitude less efficient than mitoxantrone in its ability to cleave DNA at AP sites.

The reason for less efficient AP lyase activity of pixantrone in comparison to mitoxantrone could be a lower affinity for DNA substrate and/or a decreased rate of catalysis. To address the first possibility, we performed a Thiazole Orange (ThO) displacement assay using a 17-mer double-stranded oligodeoxynucleotide that had the same sequence as in the experiment described above, except that it contained a THF moiety instead of an AP site ( Fig. 1G ). The fluorescent output of ThO is enhanced when intercalated in duplex DNA and thus, its fluorescent properties serve as a sensitive readout of the ability of other molecules to displace it from duplex DNA, thereby reducing its fluorescent signal (Boger and Tse, 2001; Simeonov et al., 2009) . As expected, both mitoxantrone and pixantrone could displace ThO from THF-containing DNA ( Fig. 1H ). The apparent DNA binding constants of mitoxantrone and pixantrone were 83 ± 3 nM and 1.25 ± 0.32 μM, respectively. Thus, pixantrone has significantly lower affinity for THF-containing DNA than mitoxantrone, which may contribute to its decreased efficiency in catalyzing incision reactions at AP sites.

Our prior study suggested that mitoxantrone inhibits the DNA repair functions of APE1 via strong, competitive binding to APE1 substrates, including AP, THF, and 3′ α,β-unsaturated aldehyde sites (Minko et al., 2024) . Given the above observations, we hypothesized that pixantrone may also interfere with APE1 endonuclease activity, but with much lower potency. To address this hypothesis, the IC _50_ of APE1 inhibition by pixantrone was measured using a plate reader-based approach with TAMRA-labeled AP site-containing oligodeoxynucleotides ( Figure 1I ), as was previously designed for mitoxantrone (Minko et al., 2024) . Pixantrone showed an IC _50_ of 20 ± 9 μM ( Figure 1J ). For comparison, mitoxantrone inhibited APE1 activity at AP sites with an IC _50_ of ≈ 0.5 μM (Minko et al., 2024) .

In summary, although our data are consistent with the reported ability of pixantrone to form covalent complexes with AP site-containing DNA (Bellamri et al., 2023) , the AP lyase activity of this compound was significantly reduced relative to mitoxantrone and inhibition of APE1 endonuclease activity was not as effective. These differences correlate well with an ~ 15-fold lower affinity of pixantrone for THF-containing DNA as compared to mitoxantrone.

## Methods

*Preparation of DNA substrates.* Preparation of double-stranded DNA substrates and conditions for conversion of dU into an AP site using uracil DNA glycosylase were previously described (Minko et al., 2024) .

*Gel-based DNA Cleavage Assays.* AP-containing DNAs were diluted in 20 mM Tris-acetate (pH 7.9), 50 mM potassium acetate, 10 mM magnesium acetate, and 100 μg/ml BSA (CutSmart buffer from New England Biolabs Inc.). Pixantrone and mitoxantrone were diluted in dimethyl sulfoxide (DMSO). Control reactions were supplemented with DMSO. Final concentrations of reagents were 100 nM DNA, 10 μM anthracenedione compounds, and 10 % (v/v) DMSO. Reactions were incubated at 37 °C for 30 min and terminated by addition of three volumes of 95 % (v/v) formamide supplemented with 10 mM EDTA. DNAs were resolved by electrophoresis and analyzed by the FluorChem M imager (Protein Simple) using a 534 nm LED light source and 593 nM emission filter as previously described (Minko et al., 2024) . The reported data represent the mean percent values ± standard deviations calculated from three independent experiments.

*ThO Displacement Assay.* ThO displacement assays were performed as previously described (Simeonov et al., 2009) with minor modifications. Reactions were conducted in 20 mM Tris-HCl buffer (pH 7.4) containing 100 mM KCl and 0.01 % (v/v) Tween-20. THF-containing double-stranded oligodeoxynucleotides were combined with ThO and pipetted into wells of a 384-well black plate. Serially-diluted mitoxantrone or pixantrone was added and following incubation for 10 min at room temperature, the fluorescence signal was measured in a TECAN INFINITE M NANO plate reader using a 480 nm (9 nm bandwidth) excitation filter and a 530 nm (20 nm bandwidth) emission filter. The reaction volume was 20 μl. Final concentrations of DNA and ThO were 50 nM and 250 nM, respectively. To correct for background fluorescence, data were collected in parallel for the reactions containing no DNA. Following subtraction of these values, the intensity of signal for each concentration of mitoxantrone or pixantrone was normalized against control reactions containing DNA and ThO only. The normalized data were plotted as a function of compound concentrations using KaleidaGraph software and the ThO displacement constants were obtained from the best fit of the data to a sigmoidal equation. The mean constants ± standard deviations were calculated from three independent experiments.

*Measurement of IC _50_* . The IC _50_ values were measured using DNA substrate that had a TAMRA fluorophore at the 5′ terminus of the AP site-containing strand and a black hole quencher 2 (BHQ2) at the 3′ terminus of the complementary strand. The DNA substrate was diluted in CutSmart buffer and distributed into wells of a 384-well black plate. Serial dilutions of pixantrone were prepared in DMSO and added to DNA using a multi-channel pipette. Reactions were pre-incubated at room temperature for 10 min prior to addition of APE1 and monitored at 37 °C in a TECAN INFINITE M NANO plate reader with fluorescence readings recorded every 2 min for 1 h. The TAMRA fluorescent signal was recorded using a 525 nm (9 nm bandwidth) excitation filter and a 598 nm (20 nm bandwidth) emission filter. Final concentrations of reagents were 50 nM DNA, 0.00125 units/μl APE1, and 10 % (v/v) DMSO. Pixantrone concentrations ranged from 0.195 to 100 μM. To account for pixantrone-catalyzed cleavage of DNA substrate, reactions were also conducted using the same pixantrone concentrations, but in the absence of APE1. All reactions were conducted in duplicates. The fluorescent signal from duplicate reactions was averaged at each time point and initial rates were obtained by fitting the linear portion of the experimental curve to a linear equation. The rates of APE1-catalyzed DNA incision were calculated by subtraction of the rates of reactions containing pixantrone only from the rates of corresponding reactions containing both APE1 and pixantrone. Relative activity was calculated as the ratio between reaction rates containing pixantrone and APE1 and the control reaction containing only APE1 and plotted against pixantrone concentrations using KaleidaGraph software. The IC _50_ values were obtained from the best fit of the data to a sigmoidal equation. The mean IC _50_ value ± standard deviation was calculated from three independent experiments.

## Reagents

Enzymes and chemicals were purchased from the following sources: Uracil DNA glycosylase and human APE1 from New England Biolabs Inc., mitoxantrone and pixantrone from Cayman Chemicals (catalog # 14842 and 20055), and ThO from Sigma-Aldrich (catalog # 390062). Oligodeoxynucleotides were synthesized by Integrated DNA Technologies (Coralville, IA). These included: (1) 5′-TAMRA-labeled 17-mer containing an internal deoxyuridine (5′-TAMRA-TCACC(dU)TCGTACGACTC-3′); (2) 17-mer containing an internal THF (5′-TCACC(THF)TCGTACGACTC-3′); (3) complementary 17-mer (5′- GAGTCGTACGACGGTGA-3′); and complementary 17-mer conjugated with BHQ2 at its 3′ terminus (5′- GAGTCGTACGACGGTGA-BHQ2-3′).
